# Sustained spatial attention accounts for the direction bias of human microsaccades

**DOI:** 10.1038/s41598-020-77455-7

**Published:** 2020-11-26

**Authors:** Cheng Xue, Antonino Calapai, Julius Krumbiegel, Stefan Treue

**Affiliations:** 1 Cognitive Neuroscience Laboratory, German Primate Center, Goettingen, Germany; 2 Leibniz-ScienceCampus Primate Cognition, Goettingen, Germany; 3 Faculty of Biology and Psychology, Goettingen University, Goettingen, Germany; 4 Department of Neuroscience and Center for the Neural Basis of Cognition, University of Pittsburgh, Pittsburgh, PA 15213 USA

**Keywords:** Saccades, Oculomotor system, Visual system, Neuroscience, Cognitive neuroscience, Attention, Cognitive control, Perception

## Abstract

Small ballistic eye movements, so called microsaccades, occur even while foveating an object. Previous studies using covert attention tasks have shown that shortly after a symbolic spatial cue, specifying a behaviorally relevant location, microsaccades tend to be directed toward the cued location. This suggests that microsaccades can serve as an index for the covert orientation of spatial attention. However, this hypothesis faces two major challenges: First, effects associated with visual spatial attention are hard to distinguish from those that associated with the contemplation of foveating a peripheral stimulus. Second, it is less clear whether endogenously sustained attention alone can bias microsaccade directions without a spatial cue on each trial. To address the first issue, we investigated the direction of microsaccades in human subjects while they attended to a behaviorally relevant location and prepared a response eye movement either toward or away from this location. We find that directions of microsaccades are biased toward the attended location rather than towards the saccade target. To tackle the second issue, we verbally indicated the location to attend before the start of each block of trials, to exclude potential visual cue-specific effects on microsaccades. Our results indicate that sustained spatial attention alone reliably produces the microsaccade direction effect. Overall, our findings demonstrate that sustained spatial attention alone, even in the absence of saccade planning or a spatial cue, is sufficient to explain the direction bias observed in microsaccades.

## Introduction

Microsaccades are small ballistic eye movements that occur even while maintaining gaze fixation ^1^. They have long been considered a product of noise in the oculomotor system^2^ until research within the last two decades revealed the relation between microsaccades and cognitive processes^3–5^. Human psychophysical studies have shown, that around 300 ms after subjects are instructed by a symbolic spatial cue (e.g. an arrow-head at gaze location, a pre-assigned color or a sound source) to attend to a certain location, the directions of microsaccades are biased toward the location indicated by the cue^6–8^. This suggests that microsaccades can serve as an index for covert spatial attention. However, such an attention-specific interpretation of the post-cue microsaccade direction bias faces two challenges. First, microsaccades share the same oculomotor generation circuitry with that of large saccades^9,10^. The planning of saccades is also known to interfere with the dynamics of microsaccades^8^. Therefore, instead of spatial attention, a more parsimonious cause for the microsaccade direction bias might simply be the contemplation of foveating a peripheral location of interest, which typically is also the location of attention^11,12^. Thus, to assess the role of attention in the microsaccade direction bias, it is important to take the subjects’ subsequent saccade target into account. Secondly, it is unclear whether the microsaccade direction bias is a reliable index of sustained attention, or, alternatively, merely a transient effect^13^. Specifically, previous studies that report the post-cue microsaccade direction bias have investigated microsaccades that shortly followed a spatial cue (typically in the same trial, ranging from 300 ms to several seconds^3,6,8,13,14^). Little to no evidence is available regarding whether this effect would last as long as spatial attention is endogenously deployed without being briefly preceded by a spatial cue. Some studies even found that for a certain time period a little later than the 300 ms window, microsaccades are directed away from the cued location^8,13^, possibly reflecting an inhibition of return^15^.

**Figure 1:**
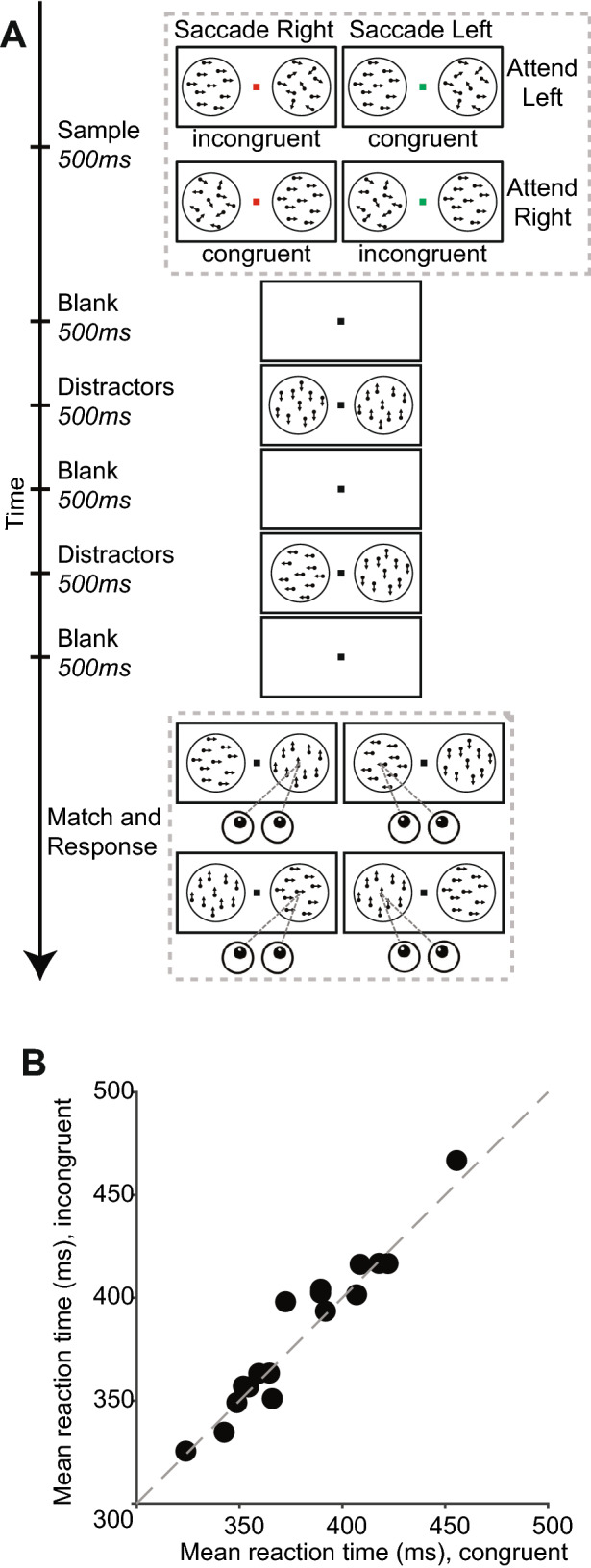
Match to sample task to dissociate attention and saccade planning. **(A)** Task flow. Once the subject pressed a button and foveated the central fixation point. One fully coherent RDP and one non-coherent RDP were displayed. The coherent RDP is the sample stimulus. After a brief blank interval, a series of stimuli-pairs followed, and the subjects needed to respond when they found a match with the sample and otherwise maintain fixation. The match can occur in any stimuli-pair at the same location as the sample, or in a small fraction of trials, does not appear at all. When the subjects found the match, they have to respond by making a saccade to one of the stimulus locations, which was instructed by the color of the fixation dot during the sample phase (red for rightward saccade, green for leftward saccade). **(B)** Mean reaction times of incongruent hit trials (when the match appeared and the subjects correctly responded) plotted against that of congruent hit trials. Each dot represents one human subject. The dashed diagonal line indicates unity line.

To address these challenges, we recorded human eye movements during periods of fixation while the subjects performed a spatial attention guided *match-to-sample* task (Fig. 1A). Our results demonstrate a consistent spatial attention effect on microsaccade directions, even without a spatial cue. These findings revealed that microsaccades directly reflect an organism’s internal attentional state, rather than oculomotor responses to visual stimuli or byproducts of saccade intentions.

## Methods

### Experiment setup

For both experiments, participants were seated 57 cm from a 22” Samsung SyncMaster 2233R monitor, operating at a resolution of 1680 × 1050 pixels and a 120 Hz refresh rate. Eye position was acquired with an SR Research Eyelink 1000 (Version 4.56) while each subject’s chin rested on a platform to maintain head position throughout the experimental sessions. The open-source software MWorks (Version 0.5, https://mworks.github.io/) was used to run the tasks and to record the subjects’ behavioral data.

### Human subjects

This study recruited 35 naïve subjects (16 for experiment 1, 19 for experiment 2, see supplementary table 1 for their gender, age, handedness, and vision profiles). The study was approved by the Ethics Committee for experiments with humans of the Georg-Elias-Müller-Institute of Psychology, University of Göttingen, and followed the principles of the Declaration of Helsinki. Informed consent was obtained from the subjects. Each subject received verbal and written information about the task, and received a small monetary compensation for his/her participation.

### Experiment 1

In experiment 1 (Fig. 1A), subjects depressed a button on a game pad (Logitech Inc., Precision) to start a trial. Each subject needed to correctly perform 480 trials to complete the experiment. During each trial, subjects were required to maintain eye fixation at a central dot (diameter: 1° of visual angle, luminance: 5.65 cd/m^2^, fixation window 2° in radius) until they made a saccade to the target location. Other fixation breaks would terminate the trial, which would be repeated later. Upon trial start, the fixation dot took on a color (either red or green) that informed the subjects whether to make a right- or leftward saccade at the end of the trial. During the sample phase, one random dot motion pattern (RDP, 8° diameter; dot luminance: 30.09 cd/m^2^, number of dots: 100, dot diameter: 0.25°, speed 5°/s) was displayed in each visual hemifield (RDPs centered at 15° eccentricity). One RDP is behaviorally relevant (the sample): it reveals the spatial location and motion direction (up, right, down, left; 100% coherence) of the target; the other RDP is behaviorally irrelevant (0% coherence): it is designed to offset the effect of microsaccade inhibition from a peripheral stimulus^16^. The sample phase was followed by up to three alternating blank periods and displays of fully-coherent RDP-pairs. Subjects were required to report a RDP with the same motion direction as the sample (a *match* stimulus), which might appear in any stimulus display period. In 10% of the trials, none of the three stimulus display periods contained a match, in which case the subjects had to withhold a response until the end of the trial. The match, if it appeared, would always be at the same location as the sample. To report a match-detection, the subjects needed to make a saccade (either leftward or rightward) according to the color (green or red) of the fixation dot during the sample phase. Thus, in half of the trials the correct saccade endpoint and the attended location were aligned (congruent trials), while in the rest the correct saccade endpoint was opposite of the attended location (incongruent trial). Upon detecting a match, the subjects were required to respond within a time window individually determined for each subject through a prior staircase procedure. During this procedure the response time window adapted after each trial, until a given subject’s performance stabilized at 80%.

### Experiment 2

The task in experiment 2 was similar to experiment 1 except for two major aspects: (1) the trials were performed in blocks of incongruent trials. Each block ends when the subject correctly performed 80 trials. Within each block, all trials had a fixed location of attention (left or right), and a fixed goal for response-saccades (always opposite the location of attention); (2) the sample phase contained only one fully-coherent sample stimulus located at the center. The location of attention (left or right) was given by a verbal instruction before each trial-block; the goal for the response-saccade was not cued in each trial since all trials were incongruent trials. In other words, no stimulus during a trial block was spatially informative in any way. Stimuli used in experiment 2 were similar to that of experiment 1: fixation dot diameter: 0.5°, luminance = 59.91 cd/m^2^; RDP 8° diameter; dot luminance: 30.09 cd/m^2^, number of dots: 100, dot diameter: 0.15°, speed 4°/s.

### Microsaccade detection

We adopted the commonly used velocity threshold method described in Engbert & Kliegl (2003) for microsaccade detection. In short, we calculated the velocity for each eye at each millisecond based on the measured eye positions within a sliding time window of 8 ms. The velocity threshold for each eye is set at six times the standard deviation of all velocity magnitudes. All threshold crossing events are then compared between the two eyes, and only binocular threshold crossings are considered microsaccades^6^. We detected 10,426 microsaccades in experiment 1, and 6790 microsaccades in experiment 2. The algorithm-detected microsaccades were also visually inspected, and the start or endpoint of 643 microsaccades were manually corrected. This operation does not affect the directions of those microsaccades, which were defined as the direction of the peak velocity of the microsaccade.

This detection procedure clearly distinguished microsaccade from other smaller fixational eye movements and potential noise in the measurement (Figure S1, example microsaccade traces). The detected microsaccades showed a monotonous relationship (Pearson’s correlation coefficient = 0.94, p < 0.0001) between amplitude and maximal speed (also known as the main sequence^17^, see Figure S2). We also observed that after a stimulus change (e.g. the offset of a stimulus), the rate of microsaccades temporarily drops, and rises to a peak at around 250–300 ms after the stimulus change (Figure S3). This is consistent with what has been reported for the dynamics of microsaccade rate^6–8^.

To investigate the microsaccade-directional profile while subjects are expecting a potential upcoming match, most results are based on microsaccades that occurred during the blank periods (except in Fig. 3, where direction profiles during stimulus periods were compared with those during blank periods).

## Results

To investigate the attentional modulation of microsaccade directions, we designed a spatially guided match to sample task (Fig. 1A), in which subjects needed to wait until a remembered stimulus appears at one of two locations. We compared the percentage of microsaccades directed to the left hemifield when attention was allocated to the left or right hemifield; and whether such an effect is contingent on saccade planning or spatial cuing.

### Task challenge: attending to somewhere while ready to look elsewhere

To disentangle the effects of the attended location and the response saccade target on the direction bias of microsaccades, two independent spatial cues were shown at the beginning of each trial in experiment 1: the attention cue, indicating the location of the match (left or right of the fixation point), and the saccade cue, instructing the goal of the response-saccade (left or right). Both cues were randomly combined for each trial, indicating either the same location (congruent trials) or opposite locations (incongruent trials). By dividing the trials either according to the location of spatial attention or the goal of the response saccade, the influences of the attended location and the response saccade target on microsaccade direction can be separately evaluated.

A critical objective of our experimental design is to encourage the subjects to already plan a saccade to a given location before the match appears (while also attending to an independent location), rather than to plan the saccade only after match detection (when the attended location is no longer relevant). Given that the subjects are under time pressure to respond as quickly as possible (see methods), the latter strategy would likely lead to a longer reaction time in incongruent trials than in congruent trials. However, none of our 16 subjects showed significantly different reaction times between the two trial types (Bonferroni-Holm corrected rank sum test, p > 0.05 for all subjects) Fig. 1B shows the subjects’ mean reaction times for congruent trials (abscissa of the scattered dots), and for incongruent trials (ordinates of the scattered dots), respectively. There is no significant pair-wise difference across subjects (p = 0.8, Wilcoxon signed rank test), either.

### Microsaccade directions are biased toward the attended location, not the saccade goal

**Figure 2:**
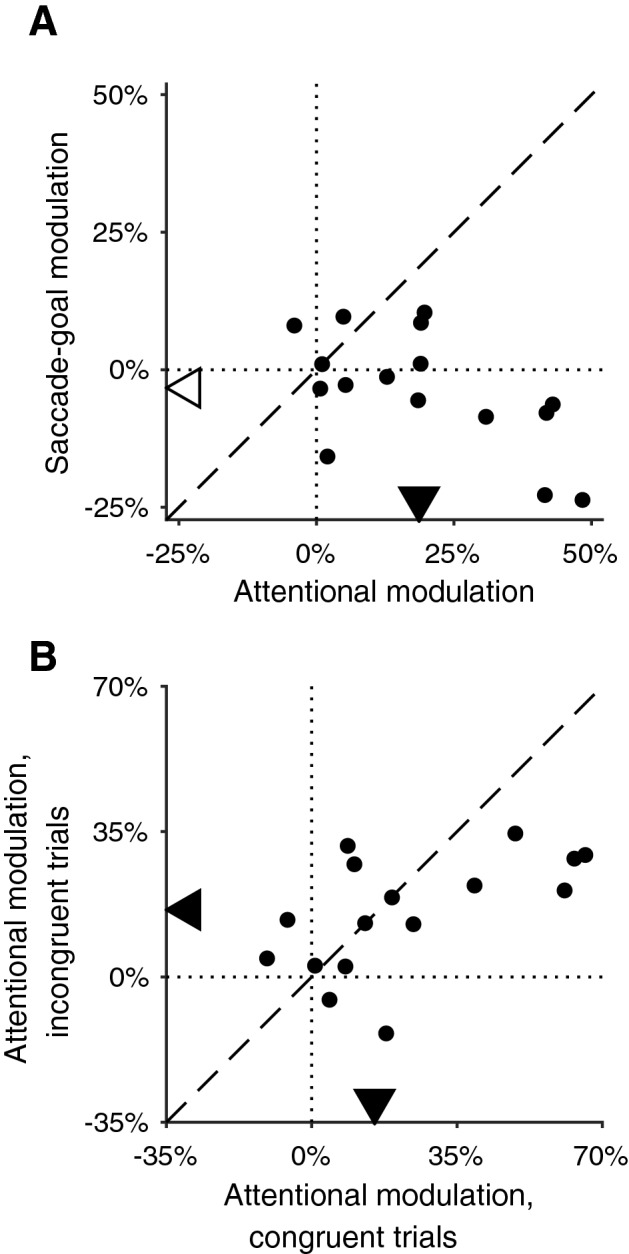
Overall microsaccade-directional modulation (see “Material and Methods”). **(A)** The microsaccade-directional modulations by attended location (abscissae) plotted against microsaccade-directional modulations by saccade goal (ordinates). Each dot represents one subject. For both attended location and saccade goal, a positive modulation indicates a microsaccade-directional bias towards the respective location. Dotted vertical and horizontal lines indicate the zero line of abscissa and ordinates, respectively. Dashed line shows the unity line. Arrows on horizontal and vertical axes indicate the median of abscissa and ordinates among all subjects, respectively. Filled symbols indicate the median is significantly different from zero; while open symbols, not. **(B)** The microsaccade-directional modulation by attention for congruent-cue trials (abscissa) plotted against that for incongruent-cue trials (ordinates). Lines and symbols are similarly defined as in A).

To obtain a quantitative measure for the magnitude of attentional modulation of microsaccade directions, we compared the distributions of microsaccade-directions during *attend left* trials and *attend right* trials for each subject. For the difference of these two proportions of the two trials types a positive attentional modulation indicates a bias of microsaccade directions towards the attended location, while a negative modulation indicates a bias away from it. Figure 2A plots the two modulations of microsaccade directions for our 16 subjects. The data clearly show that the directions of microsaccades are biased towards the attended location (median difference 18.7%, p = 0.0009, Wilcoxon signed-rank test). Overall, these attention oriented microsaccades during the blank periods shifted the gaze location away from the fixation point (Figure S4A).

Similarly, we determined the modulation of microsaccade directions by saccade-goal locations (ordinate in Fig. 2A), taking the difference of leftward-microsaccade proportions of trials requiring leftward vs. rightward response saccades. Comparing these two modulations we find that the attentional modulations are significantly larger than saccade-goal modulations, both toward the saccade-goal (attentional modulations vs. saccade-goal modulations, p = 0.003, Wilcoxon pairwise signed-rank test), and away from the saccade goal (comparing attentional modulations and the reversed saccade-goal modulations, p = 0.003). We did not observe any significant effect of the planned saccade goal on the direction of microsaccades (median difference − 3%, p = 0.3) and also no significant correlation between attentional modulations and saccade goal modulations (Kendall’s rank correlation = − 0.283, p = 0.1).

### Attentional effect on microsaccade direction is consistent for congruent and incongruent cue trials

Even though our data show that the target of the response saccade is not a significant modulatory factor of microsaccade directions, it could still have a significant interaction with attention. We therefore looked at the attentional modulations in congruent-cue trials (attention cue and saccade cue at the same location, abscissa in Fig. 2B) and incongruent-cue trials (attention cue and saccade cue at opposite locations, ordinate in Fig. 2B), respectively. Similar attentional modulations were observed in both trial types (congruent-cue trials, median difference 15.4%, p = 0.003; incongruent-cue trials, median difference 16.0%, p = 0.003; Wilcoxon signed-rank test), with no significant difference between them (p = 0.2, Wilcoxon signed-rank test). We also performed a two-way ANOVA on the percentages of microsaccades directed to the left hemifield in all four combinations of attention and saccade-goal locations, which confirmed the above conclusions: attended location is a significant factor (p < 0.0001), while saccade-goal is not (p = 0.8), and neither is the interaction between attention and saccade goal (p = 0.4).

### Attention modulates microsaccade-directional modulations during blank period and stimulus display period differently

**Figure 3:**
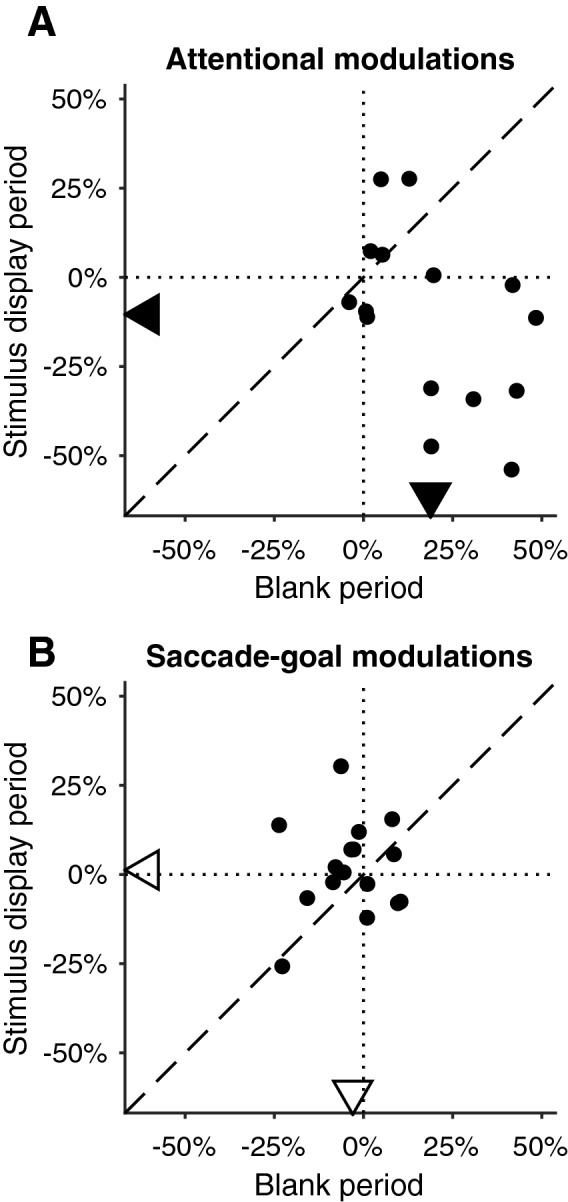
Microsaccade-directional modulations during blank periods versus stimulus display periods. **(A)** shows the modulations by attended location, **(B)** shows the modulations by saccade goal. In both **(A)** and **(B)**, each dot represents one subject; its abscissa and ordinate represent the microsaccade-directional modulations during blank periods and during stimulus display periods, respectively. Dotted vertical and horizontal lines indicate the zero line of abscissa and ordinates. Dashed line shows the unity line. Arrows on horizontal and vertical axes indicate the median of abscissa and ordinates among all subjects, respectively. Filled symbols indicate the median is significantly different from zero; while open symbols, not.

Our data show that attention biases microsaccade directions towards the attended location when subjects are expecting the onset of a potential match, as shown by the abscissas in Figs. 2A and 3A. Interestingly, however, when we look into microsaccade-directions during the display of RDP-pairs (i.e. Figure 1A distractor periods, during which the subjects correctly maintained fixation), as shown by the ordinate of Fig. 3A, microsaccade-directions were biased away from the attended location (median difference − 10.3%, p = 0.04, Wilcoxon signed rank test). This opposite effect is likely a result of gaze correction back to the fixation point, since microsaccades during this period brought gaze positions towards the fixation point (Figure S4B-C). A pair-wise comparison between the attentional modulation of microsaccade-directions during stimulus display periods and those during blank periods also showed significant difference (p = 0.01, Wilcoxon signed rank test). Similarly, Fig. 3B shows the saccade-goal modulations of microsaccade directions during blank periods (abscissa) and during stimulus display period (ordinate). The saccade-goal does not have a significant effect on microsaccade directions during stimulus display periods (median difference 1.3%, p = 0.6, Wilcoxon signed rank test), not significantly different from its microsaccade-directional effects during the blank periods (p = 0.2, Wilcoxon signed rank test).

### Sustained attention, not spatial cue, modulates microsaccade-direction

**Figure 4:**
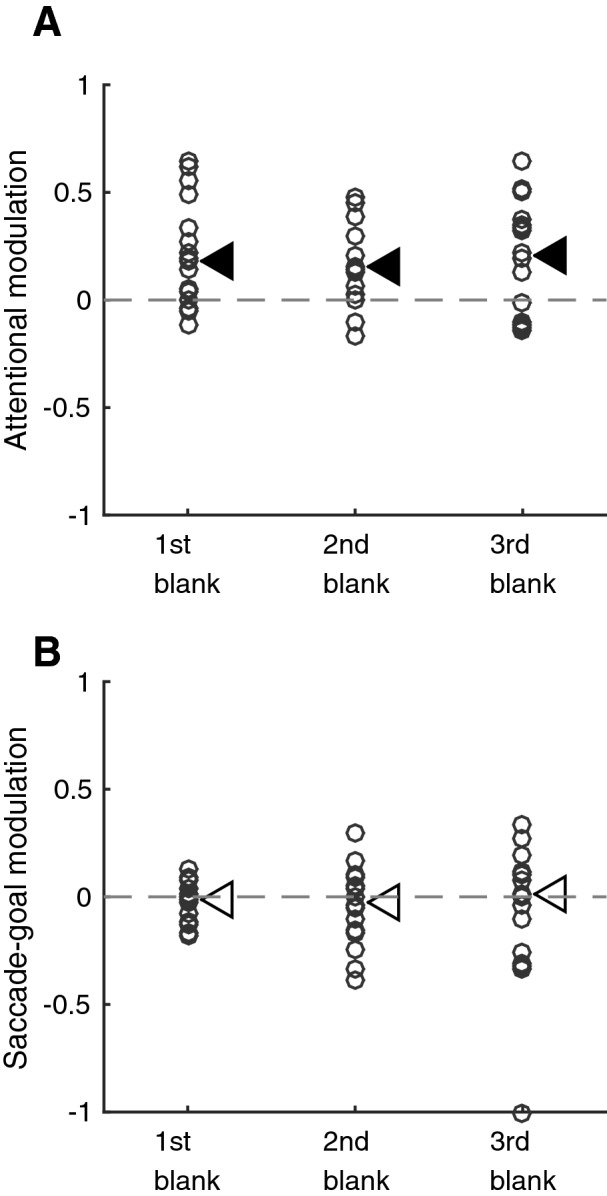
Microsaccade-directional modulations over the course of a trial. **(A)** shows the modulations by attended location, **(B)** shows the modulations by saccade goal. In both **(A,B)**, directional modulations for microsaccades that occurred during the first, second, and third blank periods are shown separately for all subjects (denoted by circles). Dashed horizontal line indicates zero modulation. Arrows indicate the median modulation during each blank period. Filled symbols indicate the median is significantly different from zero (Bonferroni-Holm corrected); while open symbols, not.

Many previous studies have reported a microsaccade direction bias shortly following a spatial cue at the beginning of every trial^1,4–6,11^. This makes it difficult to disentangle the role of sustained attention, and the role of the cue itself. In our design, the first blank period of each trial was preceded by the spatial cue (location of the sample), but the second and third blank periods were preceded with space-neutral distracting stimuli, which masked the direct visual influence from the attention cue. As shown in Fig. 4A, the attentional modulations for the three blank intervals were all positively shifted (first blank period, median difference 18.6%, p = 0.016; second blank period, median difference 15.1%, p = 0.016; third blank period, median difference 20.6%, p = 0.017; all p-values: Bonferroni-Holm corrected Wilcoxon signed rank test). This indicates that microsaccade-directions exhibit a significant bias toward the attended location, even if it is not immediately preceded by a spatial cue. Similarly, consistent with the conclusions based on all blank period microsaccades, the saccade-goal also does not significantly influence microsaccade-directions in any of the three periods alone, as shown in Fig. 4B (first blank period, median difference − 1.9%, p = 0.7; second blank period, median difference − 2.2%, p > 0.9; third blank period, median difference 0.77%, p > 0.9; all p values: Bonferroni-Holm corrected Wilcoxon signed rank test).

**Figure 5:**
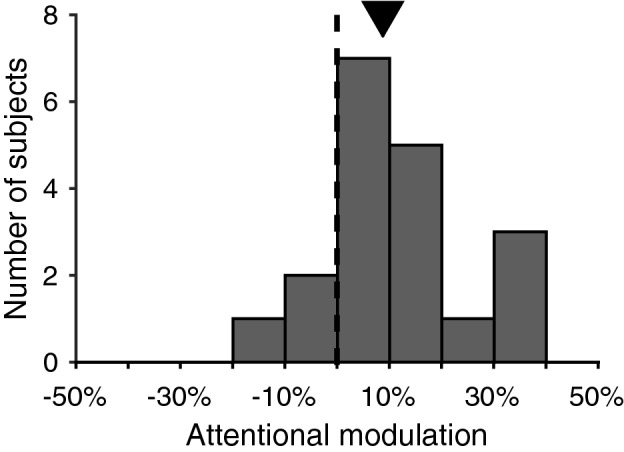
Microsaccade-directional modulations by endogenous attention, without preceding visual cue. Black vertical line indicates 0 modulation. The arrow over the histogram shows the median modulation. As in previous plots, filled symbol indicates the median is significantly different from zero.

To further isolate the microsaccade-directional modulation by sustained attention from confounds due to the visual stimulus used as a spatial cue, we conducted experiment 2, in which we tested the subjects with blocks of incongruent trials. All trials within each block had the same behavioral relevant location, and the same response saccade goal on the opposite side of the behavioral relevant location. Before each block started, we verbally provided the spatial cue, so that the trials within each block did not include a visual spatial cue. We again calculated the attentional modulation of microsaccade directions by taking the difference between the proportions of microsaccades towards the left hemifield in attend-left trial block and in attend-right trial block. The distribution of attentional modulation of microsaccade-directions is plotted in Fig. 5. We still found similar attention effects on microsaccade direction as in experiment 1 (median difference 8.9%, p = 0.003), despite the absence of a spatial cue, and induced only by the subjects’ prior knowledge of the location of the potential match. This strongly supports the hypothesis that sustained attention alone is enough to explain the bias in microsaccade direction.

## Discussion

When maintaining one’s gaze on a stationary point, small saccadic eye movements still occur. Their directions often show a bias. The cause of this microsaccade direction effect has been controversial. While there have been multiple suggestions that microsaccades can serve as an overt indicator of covert spatial attention^6–8,13,14,18–21^, others propose a dependency on other factors, such as complicated oculomotor response to visual stimuli^22–28^. A substantial set of studies has established a common neurophysiological mechanism for programming microsaccades and saccades^9,10,29,30^, which contends for the potential interaction between microsaccade generation and task required eye movements. Our study attempts to disentangle microsaccade-direction effects induced by attentional allocation from such confounding factors. Our results show that microsaccade-directions are biased towards an attended location when a subject is expecting an upcoming target at that location. We could induce this effect by sustained attention alone, even without an immediately preceding visual spatial cue. Additionally, we found no evidence for either a direct influence from oculomotor planning onto the direction of microsaccades, or an interaction between attention and oculomotor planning^21^.

Nevertheless, existing studies have sought, and failed to clearly disambiguate effects that stem from visual spatial attention and oculomotor planning on the behavioral and neuronal level^11,12,31^. Here in experiment 1 we investigated microsaccade direction bias during time periods when the subjects were required to attended to one location and contemplated a saccade to foveate an independent location, presumably at the same time. Our results provide evidence that microsaccade direction bias is contingent on covert spatial attention rather than the intention to foveate a peripheral location. It is possible, that saccade intentions do not affect microsaccade directions because when there is a conflict between attended location and intended saccade endpoint, the contemplation of the saccade is delayed until after spatial attention is disengaged from its previous location, i.e. after the match is detected. However, given that we ensure that all subjects were under similar time pressure to respond as soon as possible (see staircase procedure in “Methods”), stimulus–response compatibility would predict longer reaction times for incongruent trials than for congruent trials^32^, if oculomotor planning did not take place before the saccade go signal (the match onset). The fact that our subjects do not show such difference (either between reaction time distributions within each individual subject, or between mean reaction times across subjects) strongly suggests that oculomotor planning occurred before match onset, while the subjects were also expecting a potential match at an independent location.

As Rolfs and colleagues reported, when an exogenous cue is used to indicate one of two behavioral relevant and spatially opposite locations, microsaccades tend to be directed away from the cued locations, while an endogenous cue directs microsaccades towards the cued location. This apparent discrepancy, together with experimental observations of auditory cueing not eliciting cue-opposing bias in microsaccades directions^8^, suggests that the microsaccade-directional bias is a visual cue-related effect, rather than an attentional one. However, as Galfano and colleagues demonstrated through a paradigm designed to study inhibition of return (IOR) and attentional deployment, microsaccades directed away from a peripheral stimulus can be more simply explained as a consequence of the typical shift in spatial attention predicted by the IOR (and in accordance with reaction times patterns)^23^. The effect of visual cues is further complicated by studies where the visual cues appear in a peripheral location in one hemifield but directed attention to the other hemifield, microsaccade directions were directed towards the cue location, rather than the attended location^33^. In order to clarify such dependency, our experiment 2 inspected the microsaccade direction bias in the absence of visual cue of any form. We instructed them verbally at the beginning of each block. Despite the absence of visual cueing, the pattern of results was identical to results from our visually instructed experiment 1, namely a significant microsaccade-directional bias toward the attended location. This means the same microsaccade direction bias, usually elicited by a spatial cue on each trial, can also be elicited by endogenously sustained attention alone, through a much longer time scale (up to an hour, for our typical sessions in experiment 2). Consistent with our results, Hafed et al. showed a microsaccade direction bias up to 5 s after a spatial cue^3^. Furthermore, other studies have showed that without attentional instructions, a spontaneously occurring microsaccade also enhances perception in the direction where the microsaccade is oriented, much like the effect induced by attention^30,34^. Combined with our results, all these evidences make a very strong case that the microsaccade-directional modulation is indeed a reflection of attentional allocation, rather than a visual cue dependent effect.

Microsaccades are notoriously rare events^6–8,35^. By having alternating blank periods and stimulus display periods and adopting a big fixation point, our experiment was designed to increase the number of microsaccades per trial, to boost the statistical power of our analyses. On the other hand, one might wonder, with the relatively long trials in our experiment (up to 3.5 s) and microsaccades that are biased towards the attended location, how the subjects ensured proper eye fixation. Our control analysis in Fig. 3A showed that microsaccade-directions during stimulus-display periods were biased the opposite way to that during blank periods. This implies that microsaccades during peripheral stimulus presentations could have a distinct role: to correct eye position displacements^18,36–38^. Since a match is not expected during the time a pair of distractor RDPs are still on display, it is conceivable that microsaccades during these periods reflect the oculomotor system’s effort to countermand a fixation break after the sudden onset of peripheral RDP-pairs^15^. However, previous research showed an oscillation in microsaccade directions^15^ that in our task synchronized with the rhythmic on and off stimulus pattern in our task. To best address the role of microsaccades during stimulus display, future studies need to introduce stimulus-onset asynchrony to dissociate effects of stimulus-onset and potential confounding effects from expectation of future events.

Based on findings from human psychophysics to monkey neurophysiology^11,39–41^ there is a debate as to whether saccade preparation and visual spatial attention share the same neuronal mechanism. Our results show: (1) incongruent trials do not take longer to complete than congruent trials; (2) when subjects are instructed to maintain spatial attention and also prepare for a saccade, microsaccade directions are consistently biased towards the attended location, regardless of the saccade goal location. The former suggests that maintaining spatial attention and oculomotor planning can be executed at the same time toward different spatial locations, while the latter suggests that deploying spatial attention, but not intention to foveate a peripheral location, effects microsaccade directions. These findings argue for independent neuronal circuitries for visual spatial attention and saccade intention.

## Supplementary information

Supplementary information

## Data Availability

The datasets generated during and/or analyzed during the current study are available from the corresponding author on reasonable request.
