# Supplementary material for: Sustained spatial attention accounts for the direction bias of human microsaccades
Source: Sci Rep. 2020 Nov 26;10:20604. doi: 10.1038/s41598-020-77455-7 (PMC7692503; doi:10.1038/s41598-020-77455-7)
Supplement: Supplementary file 1 — Supplementary information [file 41598_2020_77455_MOESM1_ESM.docx]

Sustained spatial attention accounts for the direction bias of human microsaccades

Cheng Xue^1,4,*^, Antonino Calapai^1,2,*^, Julius Krumbiegel^1,3^, Stefan Treue^1,2,3^

1 Cognitive Neuroscience Laboratory, German Primate Center, Goettingen, Germany

2 Leibniz-ScienceCampus Primate Cognition, Goettingen, Germany

3 Faculty of Biology and Psychology, Goettingen University, Goettingen, Germany,

4 Department of Neuroscience and Center for the Neural Basis of Cognition, University of Pittsburgh, Pittsburgh, PA, USA

^*^ These authors contributed equally to this work.

**Supplementary material**

Supplementary table 1 : subject meta-information.

Experiment 1. Note that handedness has been assessed verbally.

| Subject | Age | Gender | Handedness | Vision |
| --- | --- | --- | --- | --- |
| ANH | 22 | f | right | normal |
| CAM | 20 | f | right | normal |
| INB | 21 | f | right | contact lenses |
| INN | 22 | f | right | normal |
| JOD | 23 | m | right | normal |
| LEI | 22 | f | right | normal |
| MAL | 26 | m | right | normal |
| MIS | 23 | f | right | normal |
| AGN | 23 | f | right | glasses |
| SVE | 23 | f | right | normal |
| ANM | 24 | m | right | glasses |
| GAE | 22 | f | right | normal |
| ALN | 20 | f | right | normal |
| THR | 23 | f | right | normal |
| LUK | 20 | m | right | normal |
| MAG | 27 | f | right | normal |

Experiment 2. Note that handedness has been assessed verbally.

| Subject | Age | Gender | Handedness | Vision |
| --- | --- | --- | --- | --- |
| FHA | 23 | m | right | normal |
| JKI | 20 | m | right | normal |
| MAS | 25 | m | left | contact lenses |
| JAK | 24 | f | right | contact lenses |
| PAK | 24 | f | right | contact lenses |
| LUB | 25 | f | right | normal |
| REP | 23 | m | right | glasses |
| KES | 25 | f | right | normal |
| SIL | 33 | f | right | normal |
| TEF | 25 | f | right | normal |
| RIW | 23 | f | right | normal |
| JUG | 23 | f | left | normal |
| DIT | 22 | f | left | contact lenses |
| CAR | 21 | f | right | contact lenses |
| SGO | 21 | f | right | normal |
| ANV | 23 | f | right | glasses |
| JMA | 31 | m | right | glasses |
